# A neglected conceptual problem regarding phenotypic plasticity's role in adaptive evolution: The importance of genetic covariance and social drive

**DOI:** 10.1002/evl3.251

**Published:** 2021-08-23

**Authors:** Nathan W. Bailey, Camille Desjonquères, Ana Drago, Jack G. Rayner, Samantha L. Sturiale, Xiao Zhang

**Affiliations:** ^1^ School of Biology University of St Andrews St Andrews KY16 9TH United Kingdom; ^2^ Department of Biological Sciences University of Wisconsin–Milwaukee Milwaukee Wisconsin 53201; ^3^ Current Address: Department of Biology Georgetown University Washington DC 20057

**Keywords:** Adaptation, indirect genetic effects, interacting phenotype, phenotypic accommodation, pleiotropy, social drive

## Abstract

There is tantalizing evidence that phenotypic plasticity can buffer novel, adaptive genetic variants long enough to permit their evolutionary spread, and this process is often invoked in explanations for rapid adaptive evolution. However, the strength and generality of evidence for it is controversial. We identify a conceptual problem affecting this debate: recombination, segregation, and independent assortment are expected to quickly sever associations between genes controlling novel adaptations and genes contributing to trait plasticity that facilitates the novel adaptations by reducing their indirect fitness costs. To make clearer predictions about this role of plasticity in facilitating genetic adaptation, we describe a testable genetic mechanism that resolves the problem: genetic covariance between new adaptive variants and trait plasticity that facilitates their persistence within populations. We identify genetic architectures that might lead to such a covariance, including genetic coupling via physical linkage and pleiotropy, and illustrate the consequences for adaptation rates using numerical simulations. Such genetic covariances may also arise from the social environment, and we suggest the indirect genetic effects that result could further accentuate the process of adaptation. We call the latter mechanism of adaptation *social drive*, and identify methods to test it. We suggest that genetic coupling of plasticity and adaptations could promote unusually rapid ‘runaway’ evolution of novel adaptations. The resultant dynamics could facilitate evolutionary rescue, adaptive radiations, the origin of novelties, and other commonly studied processes.

## The Role of Phenotypic Plasticity in Evolution

Over a century of research and scientific debate has been devoted to understanding the role of phenotypic plasticity in evolution. How might environmental influences on phenotypic expression *within* a generation affect evolutionary change *across* generations? Adaptation to variable or abruptly changed environments can be brought about through environmentally cued modifications to the phenotype, and it is uncontroversial that the plasticity of traits, i.e. the form of reaction norms, can be studied as a potentially evolvable trait in its own right (Scheiner and Lyman 1989, 1991; Via et al. 1995; Pigliucci 2001). However, plasticity has also been considered by some to be an evolutionary agent, or causal force, as plastic responses themselves influence the relative fitness of genetic variants, potentially altering both selection and responses to selection (Fusco and Minelli 2010; Radersma et al. 2020). Clearly distinguishing these characteristics of plasticity and understanding their contributions to the evolutionary process is an important conceptual challenge in modern evolutionary biology, and debating the role of plasticity in evolution has become somewhat of a subfield in its own right (e.g., Gerhart and Kirschner 2007; Mallard et al. 2018a; Svensson 2018; van Gestel and Weissing 2018; Laland et al. 2019). Here, we focus on a conceptual difficulty concerning the process by which plasticity mitigates negative pleiotropic effects of new adaptations, described below. Resolving this difficulty is central to understanding whether plasticity promotes adaptive evolution by exposing new variants to the action of selection (e.g., West‐Eberhard 1989, 2003, 2005; Lande 2009; Rajakumar et al. 2012; Levis et al. 2017) or instead hinders evolution by reducing the fitness impact of selection on traits (e.g., Huey et al. 2005; Ghalambor et al. 2007; Oostra et al. 2018). Box 1 provides a digest of the issue and our proposed model for resolving it.

**Box 1: Conceptual digest**

**A common idea**

Trait plasticity facilitates rapid adaptive evolution by offsetting indirect negative fitness consequences of new adaptive variants under selection.

**The problem**

Recombination and other processes should sever genetic associations between new adaptations and plastic traits that compensate their negative pleiotropy.

**A resolution**

Genetic covariance between new adaptive variants and plastic traits that facilitate them potentiates plasticity's role in rapid adaptive evolution.

**Conditions of this model**

(1) New variants under selection exert pleiotropic effects.

(2) Plastic traits offset negative pleiotropy of an adaptation.

(3) This facilitating plasticity is heritable.

(4) Plasticity and the adaptive trait genetically covary.

(5) *Social drive* can occur when the plasticity is cued by pleiotropic changes to the social environment.

**Testable predictions**

(1) We should detect more cases of plasticity facilitating adaptation when the plastic traits exist at high frequency prior to the emergence of adaptive variants.

(2) The rate of adaptive evolution will increase in scenarios following this order: conditions 1–3 are met < conditions 1–4 are met < conditions 1–5 are met.

## A Neglected Conceptual Challenge

The argument is commonly made that plastic adjustment of phenotypes can facilitate adaptive evolution by mitigating negative effects of new variants. This process is akin to West‐Eberhard's concept of phenotypic accommodation: “adaptive adjustment, without genetic change, of variable aspects of the phenotype following a novel input during development” (West‐Eberhard 2005). Without loss of generality and for simplicity, we focus on cases where such a novel input is caused by a new genetic variant. Under this model, plasticity of separate traits permits variants with selected adaptive effects, but detrimental negative effects, to persist long enough to respond to selection and spread through a population (West‐Eberhard 1989, 2003, 2005; Badyaev 2009; Zuk et al. 2014). This “buffering” process is commonly discussed in the context of developmental plasticity, although the mechanism is applicable to other forms of plasticity such as behavioral and life history plasticity. It has been studied using various terminologies and nomenclatures over the last several decades. In addition to West‐Eberhard's (2005) sensu stricto definition of phenotypic accommodation, other descriptions of this evolutionary process capture the same central idea and frequently emphasize the role of behavior, for example: “behavioral flexibility enables animals to compensate for changes in structure, physiology, etc, generated by changes at the genomic level” (Wcislo 1989), “behaviour's tendency…to facilitate the evolution of novel traits” (Zuk et al. 2014), “the role of behavioral plasticity in enabling colonizers of new environments to survive through phenotypic accommodation before adaptive evolution has time to occur” (Duckworth 2009), or the phenomenon of “novel variants … buffered by compensatory plastic responses” (Pfennig et al. 2010).

Irrespective of differences in the language used to describe plasticity‐facilitated adaptive evolution, its core feature is the idea that plasticity of developmental, physiological, or behavioral traits can mitigate otherwise harmful consequences of novel genetic variants, permitting their establishment and spread under selection. The disruptive consequences arise from negative pleiotropy, which has a well‐established influence on the likelihood of adaptive evolution (Fisher 1958; Carriere et al. 1994; Shirley and Sibly 1999; Orr 2005; Zhen et al. 2012; Chen and Zhang 2020). Variants arising de novo or introgressing into genomes may be the target of positive selection if they cause the expression of beneficial traits, but most pleiotropic effects of such variants are expected to be negative, lessening their total fitness advantage. In addition, pleiotropy itself varies and may depend on genetic background (Sikkink et al. 2015; Kingma et al. 2020). Understanding how negative pleiotropy is mitigated is a major goal of adaptation models, and has been central to understanding rapid adaptation in natural systems (Berticat et al. 2004; Wagner et al. 2008; Rostant et al. 2017). Plasticity that accommodates these effects (or “facilitates,” “compensates,” “buffers,” or otherwise mitigates them) can maintain overall organismal fitness by rebalancing trait‐level fitness effects when new variants invade the genome under selection.

Trait plasticity may mitigate negative pleiotropy in a variety of ways, depending on the developmental timing and precise effects of that pleiotropy. For example, negative pleiotropy might alter the social environment of organisms through the disruption of reproductive systems or mating signals, and plastic behavioral traits might compensate such effects to an extent that enables survival or mating within the changed social environment. Behavioral habitat choice can be considered a form of plasticity which may reduce fitness costs if an adaptive variant under selection also produces a mismatch between the organism carrying it and their immediate physical environment (Edelaar et al. 2017). Negative pleiotropy can also be more direct, affecting organismal functioning at the individual level. Individuals carrying alleles that code for plastic responses that reduce the fitness burden of such individual effects would have a selective advantage. An interesting example of how this might occur is through plastic modification of gene expression. The heat shock protein Hsp90 is illustrative. Hsp90 is a heat‐sensitive molecular chaperone that binds to unstable signal transduction proteins and deactivates them until they are conformationally stabilized and ready for deployment during molecular signaling. A study of mutations in *Drosophila melanogaster* Hsp90 famously revealed its role in suppressing the expression of cryptic genetic variation (Rutherford and Lindquist 1998). By reducing expression of genetic variants that exert considerable harm if active at an inappropriate spatiotemporal stage of development, Hsp90 mitigates negative fitness effects of those variants. Plastic molecular pathways that modify gene expression may play a similar role in filtering out negative effects of new adaptive variants. For example, one mode of action of insecticide resistance in the mosquito *Culex pipiens* is through amplification of the gene *Ester*, which results in greater esterase activity favored by selection (Raymond et al. 2001). A variety of gene amplification events have resulted in multiple resistance alleles with varying numbers of duplicate copies of *Ester*, and some of these exert more damaging negative pleiotropy than others, for example, by increasing the chances of predation (Berticat et al. 2004). Thus, if an adaptive variant overshoots an optimal level of gene expression, plastic modifiers of gene expression that can buffer such effects would facilitate the variant's evolutionary spread.

Despite the intuitive appeal of this idea, we suggest that there is a critical, neglected caveat: it is not a given that an adaptive trait and a plastic trait or traits that offset its indirect negative effects should be genetically or phenotypically associated. In contrast, it is expected that recombination, segregation, and independent assortment will quickly sever any genetic associations between them. In cases where trait plasticity that mitigates negative pleiotropy persists without a heritable genetic basis, plasticity may facilitate establishment of a new adaptation regardless of the genetic background that the adaptive variant invades. However, it is increasingly appreciated that there is individual, genotypic, and metapopulation‐level variation in the strength and direction of plasticity. Although there has been longstanding controversy about the genetic basis of plasticity (Via et al. 1995), it is widely accepted that plasticity can be viewed as a trait, for example, as a reaction norm, that genetic variation for plasticity exists, and that the magnitude and fitness consequences of plasticity can vary widely among genotypes (Pigliucci 2001). Genetic variation in the plasticity of traits is central to prominent models of plasticity's evolutionary causes and consequences, and empirical studies demonstrating genotypic variation in plasticity abound (Scheiner 1993; Scheiner and Goodnight 1984; Scheiner and Lyman 1989, 1991; Pigliucci 2001; Nussey et al. 2005; Aubin‐Horth and Renn 2009; Pascoal et al. 2018; Lafuente and Beldade 2019). Gene‐by‐environment interactions, for example, describe the situation in which different genotypes produce different reaction norms, that is, environmental variation affects trait expression differently for different genotypes, and thus plasticity has a heritable genetic basis and can evolve (Ingleby et al. 2010, although see Arnold et al. 2019). A frequent assumption is that plasticity carries fitness costs (DeWitt et al. 1998; Auld et al. 2010). Plasticity is therefore not expected to be uniform across all genetic backgrounds present in a population, and may even evolve in the absence of environmental heterogeneity (Matthey‐Doret et al. 2020).

Genetic variation in plasticity is important, because for plasticity to play a role in facilitating the evolution of novel traits in the manner described above, individual genotypes expressing such traits must also produce appropriate plastic responses to their altered circumstances (Duckworth 2009). Unless facilitating plasticity exists without a heritable genetic basis, any such genetic association is also subject to decay through the action of recombination and random assortment. Adaptive genetic novelties do not necessarily arise or introgress into a genetic background coding for facilitating plasticity. How, then, does a situation arise in which plasticity effectively facilitates the rapid evolution of novel adaptations? Resolving this conceptual challenge is necessary to accurately evaluate the general importance of plasticity's role in adaptive evolution.

## Resolution: Genetic Covariance of Adaptive Traits and Plasticity

**Figure 1:**
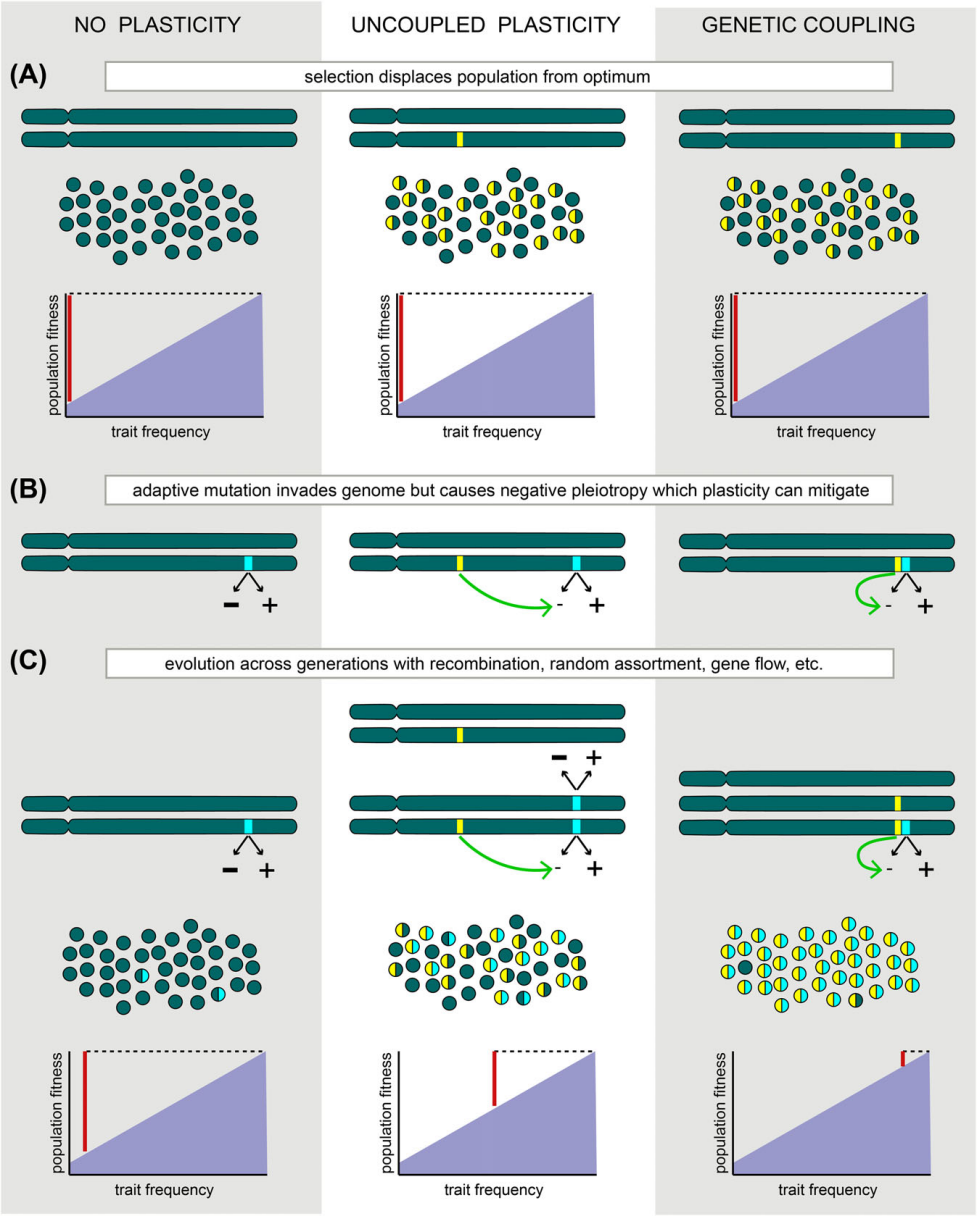
Simplified illustration depicting the influence of genetic architecture on plasticity‐facilitated adaptation. All three columns depict a population exposed to new selection. The left column represents a scenario without preadapted facilitating plasticity. The middle represents a population in which preadapted alleles exist that cause facilitating plastic trait expression (yellow bars) but these alleles do not covary with a de novo adaptive variant (blue bar). The right column illustrates a scenario in which preadapted alleles conferring trait plasticity that mitigates negative pleiotropy are physically linked with an adaptive variant, and therefore strongly genetically covary. Colored circles show the hypothetical distribution of haplotypes in each population, where shading indicates the background/plasticity (green/yellow) and ancestral/adaptive (green/blue) variants carried by each individual. Population‐level fitness is shown in graphs. Vertical red bars indicate each population's fitness relative to a new optimum under directional selection (shading); longer red bars indicate a more poorly adapted population. (A) Populations displaced from a phenotypic optimum due to novel selection. (B) A new adaptive variant invades the gene pool, for example via introgression or de novo mutation. It is important to note that the reverse scenario is equally plausible, that is, an existing low‐frequency variant becomes adaptive when the selective environment changes (cf. Fisher 1958). The adaptive variant is assumed to have a positive impact on fitness (+), but may also cause negative pleiotropy (–) that could inhibit its spread. The effect of genetic variants (alleles, haplotypes, or multivariate genetic covariance) underlying plasticity that mitigates these negative fitness consequences is indicated by green arrows. (C) Over successive generations of selection, plasticity facilitates the spread of the adaptive mutation by reducing its negative pleiotropic consequences. However, recombination, random assortment, gene flow, and other processes can sever the association between plasticity and the adaptive trait, reducing the speed of its spread and fixation. In this simplified example, recombinant genotypes and the haplotypes in panel (C) illustrate this effect. However, if facilitating plasticity genetically covaries with the adaptive trait, and in cases where facilitating plasticity is a response to variation in the social environment (see Main Text), then runaway adaptation is predicted to result in the rapid spread or fixation of the adaptive trait. In this case, population fitness is more rapidly increased (short vertical red bar in graph on bottom right).

We propose that genetic covariance of trait plasticity that facilitates a novel adaptation, and the adaptive trait itself, resolves this problem. Assuming genetic variation in plasticity as well as in the adaptation, and considering how these genetic architectures are related, generates new predictions about plasticity's role in adaptive evolution. The foremost of these is that rapid, plasticity‐led adaptation is most likely to occur when a trait under selection genetically covaries with plasticity mitigating that trait's indirect negative fitness effects. Reaction norms are unlikely to be controlled by a single locus or particularly simple genetic architectures, but the genetic explanation shown in Figure 1 visually illustrates the conceptual challenge of recombination to models of adaptation via facilitating plasticity and the potential role of genetic covariance in overcoming it. The scenario is readily extendable to more complex multivariate quantitative genetic cases, and we use the term genetic covariance as a generic descriptor covering scenarios of plasticity‐adaptation association arising from genomic hitchhiking, genetic coupling arising from physical linkage, reduced recombination, for example, due to colocalization within inversions, supergenes, or pericentromeric chromosomal locations (Shaw et al. 2011), or multivariate genetic covariance, for example arising from gametic phase disequilibrium. The interesting case of a phenotypic covariance between adaptation and plasticity *without* a direct genetic covariance is examined in the next section.

Under this model, plasticity mitigating negative fitness effects of a new genetic variant reduces the chances that the variant will be lost due to drift, and the phenotypic association between plasticity and a novel adaptation is maintained. The likelihood or rate of adaptive evolution is therefore predicted to be enhanced by a positive genetic covariance. It is noteworthy that the direction of the covariance is relevant to evolutionary outcomes, and failure to appreciate this could cause inaccuracies in interpreting the evolutionary role of plasticity from empirical data. It is conceivable that plastic traits that adaptively mitigate negative pleiotropic effects of variants under selection could be negatively genetically correlated with those traits, such that the genetic architecture of plasticity *counteracts* its facilitating effects on evolution despite the superficial appearance of an adaptive benefit (e.g., Svensson et al. 2020). For example, if an adaptive mutation arises at a locus proximate to that of a segregating variant that influences a relevant plastic response, but in an individual who does not carry adaptive plasticity‐associated variants, then it will be less likely to be co‐expressed, inhibiting its establishment within a population. We raise this scenario as an important caveat to studies that find empirical support for adaptive, pre‐existing plasticity as a facilitator of novel trait evolution (Zuk et al. 2014).

The suggestion that plasticity could facilitate evolutionary adaptation is superficially similar to saying that two adaptive traits are better than one: separate adaptations providing a combined fitness advantage are more likely to be beneficial to an organism under selection than just one or the other. However, an important difference is that this model leaves plasticity intact after a population reaches a new optimum; genetic assimilation may occur but is not an inevitable outcome. This is consistent with recent findings that adaptive plasticity in the polychaete worm *Ophryotrocha labronica* can persist for multiple generations under strong environmental selection that might otherwise be expected to erode it (Gibbin et al. 2017). Therefore, if a plastic response mitigates negative pleiotropy of an adaptive variant during an episode of adaptation, but is not itself beneficial at the new population optimum, its expression need not become canalized (Sun et al. 2020). As an illustration of the neglected role of genetic covariance during adaptive evolution facilitated by plasticity, we are unaware of any studies that have tested for all the components of this particular genetic covariance architecture (Fox et al. 2019). To demonstrate its importance for adaptive evolution, we describe and simulate a hypothetical example in Box 2.

## Social Drive: The Role of Indirect Genetic Effects

Are there circumstances in which genetic covariance between plasticity and an adaptation is expected to drive *especially* rapid evolution? To answer this, it is useful to distinguish the evolutionary role of plasticity caused by abiotic versus social environments, because plasticity arising from the social environment is predicted to have a stronger effect on evolutionary rates due to feedback effects. Socially cued plasticity appears to be a nearly ubiquitous feature of organisms (Kasumovic and Brooks 2011). For example, studies on the evolutionary genetic model genus *Drosophila* have revealed social plasticity in traits such as aggression (Ueda and Kidokoro 2002; Saltz and Foley 2011; Saltz 2013), sexual signaling (Kent et al. 2008; Krupp et al. 2008), courtship (Schneider et al. 2017), immunity (Leech et al. 2019), and mating behavior (Marie‐Orleach et al. 2020). Such socially cued plastic changes often involve indirect genetic effects (IGEs), which describe a situation in which the phenotype of a focal individual is determined in part by the genotype of an interacting conspecific in their social environment (Moore et al. 1997). Consequently, when IGEs occur, the environment contains genes and can itself evolve and contribute to evolutionary feedback (Moore et al. 1997; Bailey 2012; Bailey et al. 2017). The resulting feedback effects can accelerate or decelerate rates of evolutionary change (Moore et al. 1997; McGlothlin et al. 2010).

**Figure 2:**
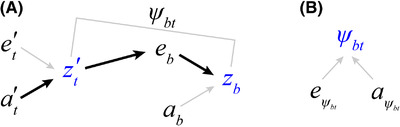
Path diagrams illustrating components of social drive via indirect genetic effects. The diagrams depict a trait‐based model, with traits indicated in blue letters and their environmental and additive genetic variances indicated with black letters. (A) The phenotype $z_{t}^{\prime }$ represents an adaptive trait that affects the social environment ($e_{b}$) and causes negative fitness consequences. Primes are used to indicate effects arising from interacting partners in the social environment. If a different trait mitigates these negative fitness consequences, such as a behavior ($z_{b}$) that allows individuals to cope with the altered social environment, there is potential for IGEs to occur. These IGEs arise from genes expressed within the social environment, and are represented by the path of solid black arrows. The interaction coefficient ($\psi_{bt}$) describes how IGEs caused by trait $z_{t}^{\prime }$ affect expression of behaviour $z_{b}$. (B) The interaction coefficient ψ, while describing effects of one trait upon the expression of another, can also be treated as a trait itself. It is well‐known to vary across genotypes (Bailey and Desjonquères unpubl. ms.) and the diagram shows how it can be partitioned into environmental ($e_{\psi_{bt}}$) and additive genetic ($a_{\psi_{bt}}$) components (cf. eq. 3 of Kazancioğlu et al. 2012). Socially cued plasticity will genetically covary with adaptive trait variants, and social drive can occur, when $|cov(a_{t}^{\prime },a_{\psi_{bt}})|>0$.

When an adaptation is genetically coupled with a plastic trait mitigating negative effects of the adaptation, and this plasticity involves IGEs, evolutionary feedback has the potential to further accelerate the pace of adaptive evolution. That is, if an adaptation affects the wider social environment, and plastic trait expression arises due to that heritable variation in the social environment, the resulting IGEs would be expected to cause dramatically enhanced evolutionary feedback effects orders of magnitude faster than evolutionary responses that do not involve IGEs (Moore et al. 1997). This is because as a novel mutation spreads, pleiotropic consequences can alter the broader social environment. Any genotypes that produce traits that are adaptively responsive to this changing social environment will have a selective advantage, and if they are genetically coupled to the novel mutation itself, coevolution of the social environment and the adaptive trait may be extremely rapid (Rubenstein et al. 2019). This can be formally studied by examining the genetic covariance between the trait under selection and the IGEs that facilitate it, expressed as $\mathrm{cov}(a_{t}^{\prime },a_{\psi_{\mathrm{bt}}})$. A full explanation of terms in this expression follows, and Figure 2 depicts the components of this genetic relationship in a path diagram. For the purposes of illustration, $z_{t}^{\prime }$ describes a phenotype for which there is an adaptive variant exerting indirect effects on the social environment. The prime indicates that it is interacting individuals carrying the adaptive variant who are causing variation in the expression of other individuals’ phenotypes. The second trait $z_{b}$ represents a phenotype whose expression is responsive to the varied social environment *caused* by $z_{t}^{\prime }$, in turn *mitigating* negative effects of $z_{t}^{\prime }$. The subscriptb is used for illustration to suggest a phenotypically plastic behavioural response to the social environment that permits individuals carrying the adaptive variant of $z_{t}$ to survive and reproduce; a real‐world example is described below. The direct additive genetic effects for each trait are indicated by a, and environmental effects by e. In IGE theory, the effect that interacting social partners have on the expression of traits in focal individuals is captured by the interaction coefficient ψ, where subscripts indicate which trait is affected by which (Moore et al. 1997; Bleakley et al. 2010; Bailey and Desjonquères unpubl. ms.). The parameter ψ is a path coefficient ranging from [−1, 1], such that its absolute value and sign indicate the strength and direction of IGEs. Thus, $\psi_{bt}$ could be interpreted as a reaction norm to genes expressed within the social environment.

The interaction coefficient ψ can be thought of as an evolvable trait in its own right (Kazancioğlu et al. 2012). In the example above, the term $a_{\psi_{bt}}$ thus represents genetic variation in facilitating behavioural plasticity, and the expression $cov(a_{t}^{\prime },a_{\psi_{bt}})$ represents genetic covariance between an adaptive trait and facilitating plasticity. The utility of this expression is that well‐established quantitative genetic frameworks have been developed to estimate all of its components (Moore et al. 1997; Bleakley et al. 2010; McGlothlin and Brodie 2009; Bijma 2010, 2014; Kazancioğlu et al. 2012; Bailey and Desjonquères unpubl. ms.). Univariate cases are readily extensible to multivariate situations where multiple interacting traits influence a focal trait, and the interactions are captured by a square matrix Ψ of interaction coefficients (Moore et al. 1997). Feedback from ensuing IGEs can cause unstable, runaway dynamics similar to the classic runaway process driving rapid evolutionary elaboration of sexual traits and preferences (Bailey and Kölliker 2019). We predict that such runaway will result in unusually rapid adaptive evolution, a process we call *social drive*.

A particularly interesting feature of social drive is that a genetic covariance between plasticity and adaptive traits that it facilitates need not be direct. That is, when phenotypic expression is changed as a result of genes in the social environment, covariance between a trait under selection and IGEs that facilitate its persistence could arise. Such an indirect covariance may occur when individuals carrying the adaptation experience a social environment that provokes the expression of plasticity that adaptively mitigates negative pleiotropy. A more concrete example of how this could happen is through a metapopulation level covariance in which subpopulations containing an adaptive variant and plastic traits are isolated from populations containing nonadaptive variant and nonadaptive, or no, plasticity. In this case, linkage disequilibrium between the adaptive trait and the plastic trait would not be detectable at the subpopulation level, but a cross‐deme positive covariance would still drive adaptation promoted by facilitating plasticity. An indirect covariance such as this might arise due to nonrandom assortment processes such as the behavioral habitat matching described above.

Evidence supporting the process of IGEs mitigating consequences of a novel trait—social drive—has been detected in the rapid evolutionary spread of silent, ‘flatwing’ crickets (*Teleogryllus oceanicus*) in Hawaii. A single‐locus genetic variant, *flatwing*, segregates on the X chromosome and causes male silence. The *flatwing* variant erases sound‐generating structures on male forewings and protects males from an endoparasitic Tachinid fly (*Ormia ochracea*) that is attracted to their song and fatally infests their body cavities. The flatwing morphotype was observed to have spread extremely rapidly, and in one population on the island of Kauai has become fixed within the span of ca. 50 generations (Tinghitella et al. 2018; Rayner et al. 2019a). Accompanying this dramatic selective sweep, however, were correspondingly radical alterations to the social environment due to elimination of the dominant sexual acoustic signal of male song (Zuk et al. 2006, 2018). Male wing morphology (i.e., ability to sing) is analogous to the trait $z_{t}^{\prime }$ in Figure 2.

There is increasing evidence that the X‐linked locus causing flatwing exerts significant pleiotropic effects in addition to the more obvious disruption to mate recognition systems (Pascoal et al. 2016, 2018; Rayner et al. 2019b; Richardson et al. 2021). In populations where flatwing has spread rapidly under selection, a variety of reproductive, behavioral, and physiological traits show plastic responses to the altered, largely silent, social environment. For example, males are more likely to adopt alternative mating tactics and behave as satellites to any remaining singers after experiencing silent conditions that mimic an all‐flatwing population (Bailey et al. 2010). Females experiencing silent conditions are more responsive and less choosy of males, suggesting relaxed mate acceptance thresholds when there is a perception of few available mates (Bailey and Zuk 2008, 2012; Bailey et al. 2008; Tinghitella et al. 2009). Male movement behavior is similarly affected by the social environment (Balenger and Zuk 2015). These plastic behavioral traits have facilitated the evolutionary spread of the flatwing morph by compensating negative effects of song loss. They are underpinned by IGEs and analogous to $z_{b}$ in Figure 2, because the presence of *flatwing* alleles in the social environment influences behavioral trait expression. Critically, one pleiotropic or hitchhiking effect of the *flatwing* locus is greater socially cued plasticity of gene expression in the brain to the variation in the social environment caused by the mutation itself (Pascoal et al. 2020). The term $cov(a_{t}^{\prime },a_{\psi_{bt}})$ developed above describes this genotypic association between socially responsive behavioral plasticity and the rapidly spreading flatwing morphotype in the Hawaiian cricket system. Thus, a direct genetic link between an adaptive variant under selection and plasticity to the subsequently altered social environment may be driving extremely rapid adaptation in this system. Empirical examples from other systems are similarly suggestive of an evolutionary process resembling social drive (Table 1).

**Table 1:** Empirical examples of components of the process by which genetic coupling of trait plasticity and adaptations drive rapid adaptive evolution. No study to our knowledge has assessed all of these simultaneously

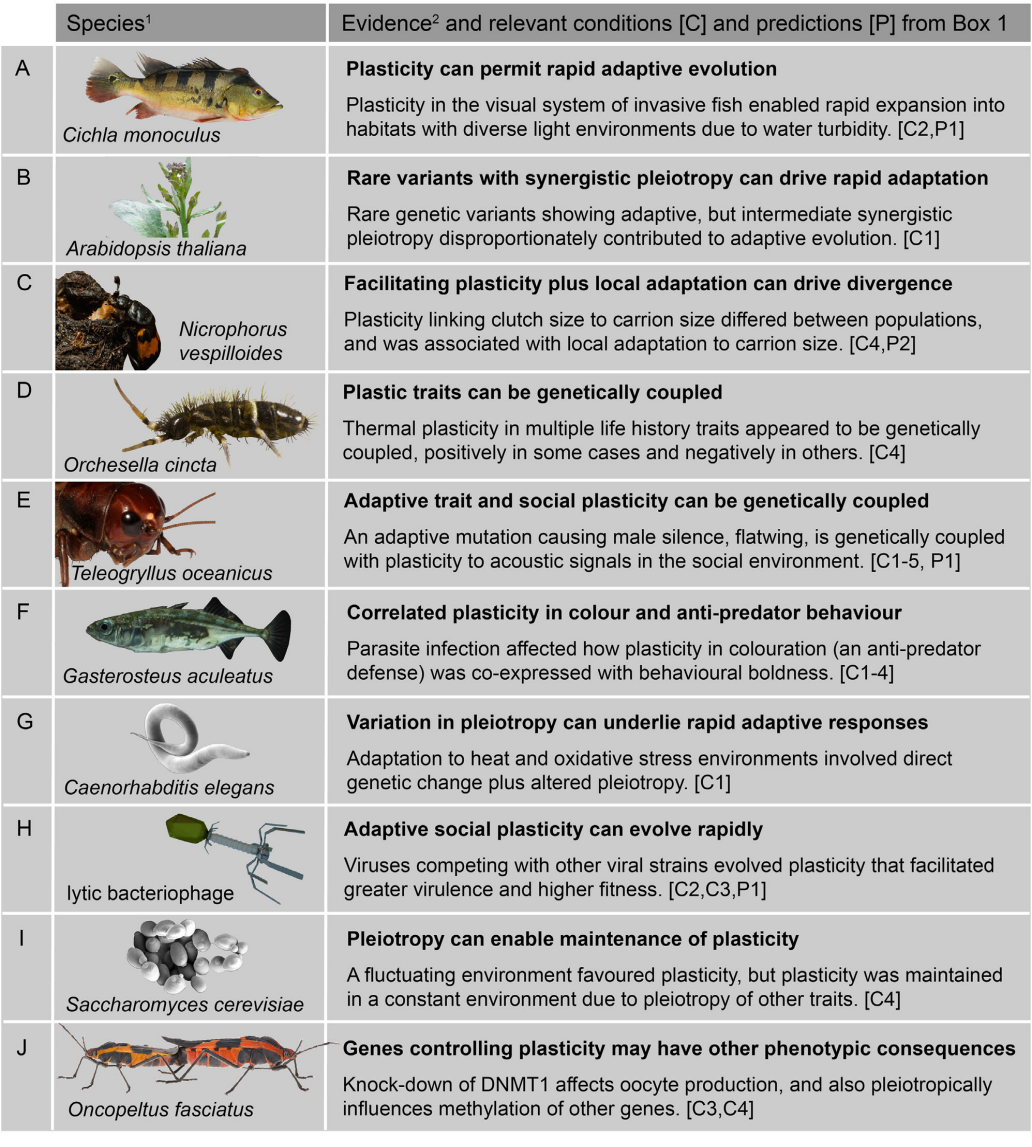

^1^Photo and illustration credits: (A) Daniel Escobar‐Carmacho; (B) Fabrice Roux; (C) Tom Houslay; (D) Theodoor Heijerman; (E) Nathan W. Bailey; (F) Petter Tibblin and Marcus Hall; (G) Arne Hendriks; (H) Baroco Ferison; (I) Mogana Das Miurtey and Patchamuthu Ramasamy; (J) Jena Johnson.

^2^References: (A) Escobar‐Carmacho et al. (2019); (B) Frachon et al. (2017); (C) Sun et al. (2020); (D) Ellers and Driessen (2010); (E) Pascoal et al. (2018); (F) Tibblin et al. (2020); (G) Sikkink et al. (2015); (H) Leggett et al. (2013); (I) Kingma et al. (2020); (J) Amukamara et al. (2020).

## Conditions, Predictions, and Research Goals

Rigorously testing the genetic architecture of adaptive traits and plasticity of separate traits that facilitates such adaptations will strengthen understanding of phenotypic plasticity's general role in evolution. We question whether the lack of strong empirical evidence for plasticity's contribution to adaptive evolution, despite it being predicted, may result from the processes described above that break down genetic associations between plasticity that facilitates adaptive traits and the traits themselves. Recent findings are increasingly solidifying the view that plasticity is genetically variable: whether studied as crossing reaction norms, molecular mechanisms (Aubin‐Horth and Renn 2009), epigenetically controlled changes in expression (Katsumura et al. 2020), or IGEs arising from the social environment (Bailey et al. 2017). It follows that plastic traits that mitigate negative pleiotropy of adaptations will more swiftly facilitate adaptive evolution in cases where the trait plasticity and the adaptation genetically covary. We assume that the initial introduction of alleles coding enhanced plasticity of a trait, for example, would be susceptible to the same sort of demographic and population genetic processes that influence loss versus maintenance of low‐frequency variants generally. Thus, if mitigating trait plasticity is pre‐existing for some reason before an adaptive variant invades, we expect this scenario to be much more favorable for plasticity to drive rapid adaptation as it avoids the risk of facilitating plasticity being stochastically lost. In contrast, if plasticity‐coding alleles and an adaptive variant invade simultaneously and are therefore both present in low frequencies, there is less chance of a positive covariance emerging unless they are physically linked. The simulation in Box 2 provides further detail about effects of these allelic starting frequencies.

Conditions predisposing a population to rapid adaptation via the mechanism we have described may not be frequently observed, but when genetic coupling does arise it should lead to distinct and rapid dynamics. Thus, as with “magic traits” in speciation (Servedio et al. 2011), we advocate testing whether such conditions are overrepresented in known cases of rapid adaptation. To our knowledge, no study has simultaneously evaluated all assumptions and predictions of the mechanism we describe. We therefore advocate testing the following conditions of our verbal model and hierarchical predictions about the pace of adaptive evolution:

**Condition 1 (Pleiotropy of adaptive variants)**: De novo or introgressed genetic variants under selection exert pleiotropic effects on other traits.
**Condition 2 (Plastic mitigation of negative pleiotropy)**: Negative pleiotropic effects of adaptive genetic variants are offset by facilitating plastic traits.
**Condition 3 (Heritable plasticity)**: This facilitating trait plasticity (i.e., reaction norm slope) has a heritable genetic basis.
**Condition 4 (Genetic coupling)**: There is genetic covariance between the plastic trait and the adaptive trait under selection.
**Condition 5 (Social drive)**: Facilitating plasticity is caused by IGEs arising from negative pleiotropic changes to the social environment.
**Prediction 1**: We should detect more cases of plasticity facilitating rapid adaptation when facilitating plastic traits are established in a population via other mechanisms prior to the emergence of adaptive variants.
**Prediction 2**: The rate of adaptive evolution will be increased in scenarios following this order: conditions 1–3 are met < conditions 1–4 are met < conditions 1–5 are met.

Dissecting the pleiotropic consequences of adaptive mutations is a priority. In insect systems, there are a number of compelling examples of de novo mutations that confer a selective advantage in the context of insecticide resistance, but with pleiotropic costs involving life history or reproductive disadvantages (Berticat et al. 2004; Rostant et al. 2017). It is impossible to capture all the pleiotropic consequences of such mutations, as these could manifest in a near‐infinite combination of tissues, behaviors, structures, and life stages. However, we may increasingly approach a fuller understanding using gene knockout panels in model systems such as *Saccharomyces cerevisiae* (Kim et al. 2009; Yadav et al. 2015) and *Drosophila melanogaster* (Massey et al. 2019). Such work is beginning to indicate how pervasive pleiotropy is, and future studies would also benefit from considering the potential for, and role of, positive pleiotropy (Rayner et al. 2019b).

Understanding the genetics of plasticity that offsets negative pleiotropy is required to falsify or support plasticity's role as an evolutionary accelerant during adaptation. Recent genotype‐by‐environment interaction studies have demonstrated how mature quantitative genetic modeling frameworks can be used to explore the evolutionary basis of phenotypic plasticity, and test the crucial prediction of genetic covariance. Although this covariance is a sufficient condition for accelerating evolution, it is not strictly necessary. The likelihood that it will enhance plasticity's role in adaptive evolution can be arranged in a rough hierarchy dictated by the robustness of the covariance to the action of recombination, random assortment, and gene flow: pleiotropy ensures the most impervious genetic covariances, followed by architectures such as inversions, or colocalization of adaptation and plasticity loci in areas of low recombination, followed by gametic phase disequilibrium. Future work would benefit from examining the stability of indirect genetic covariances driven by IGEs or cross‐deme genotypic assortment discussed above. There has also been marked progress dissecting the genetics of phenotypic plasticity in the last several decades (Aubin‐Horth and Renn 2009). A large range of mechanisms underlie the regulation of plasticity for different traits (e.g., flowering time [Li et al. 2018], morphological polyphenism [Levis et al. 2018], and behavior [Cardoso et al. 2015]), which makes it difficult to generalize about the number of loci, effect sizes, and chromosomal distribution of genes contributing to facilitating plasticity. To study genetic covariance involving plasticity, evolve‐and‐resequence studies would allow genotypes to be controlled using inbred lines, and responses to selection evaluated using whole‐genome resequencing technology (Burghardt et al. 2018; Mallard et al. 2018b). The genetic architecture and genome dynamics of adaptive traits and the plasticity that accommodates them can thus be evaluated during adaptive evolution in real time (Graves et al. 2017; Li et al. 2018; Young et al. 2018). Another topical approach is to sidestep organism‐level phenotypes such as morphology or behavior, and instead test patterns of gene expression in tissues using RNA‐sequencing technology (Ghalambor et al. 2015; Pascoal et al. 2018).

## Conclusions

The genetically explicit mechanism we describe above resolves a problem that is implicit, yet largely overlooked, in verbal models of plasticity‐facilitated evolution. Testing predictions about negative pleiotropy and the genetic architecture of facilitating trait plasticity can clarify the general role of plasticity in adaptive evolution, and can be accomplished using topical quantitative genetic and genomic frameworks. The main goals are to measure negative pleiotropic effects of adaptive genetic variants, evaluate evidence for or against genetic associations between facilitating plasticity and those variants, and test rates of adaptation when such genetic covariance occurs, when there is plasticity that facilitates adaptations but no genetic association, and without any such plasticity (cf. Fig. 1). In cases where adaptive traits alter the social environment, evolutionary feedback may occur as a result of IGEs generated by the social environment, a special case that we refer to as *social drive* and that we predict will underlie examples of exceptionally fast adaptive evolution in natural systems.

It is a truism that extant organisms we presently observe in nature harbor patterns of genetic variation, traits, and historical contingencies that favored their current adaptive fit to their environment. That means we can test the role of phenotypic plasticity in rapid adaptation by dissecting the genetic architecture of adaptations and plastic traits suspected to facilitate their spread in populations currently undergoing rapid adaptation. Genetic covariance of facilitating plasticity and adaptations may be more commonly observed than might be expected due to the dynamics that arise as a result of this genetic association favoring rapid adaptive evolution. Predicting factors that predispose populations to such adaptive processes will become increasingly important in applied contexts targeting the conservation of biological diversity, evolutionary rescue, and disease, pest, and antimicrobial resistance evolution.

**Box 2: Simulation of genetic coupling in adaptive evolution facilitated by plastic traits**

Consider a simple, hypothetical scenario. Under increasing environmental temperature, a mutation, *ht*, arises that improves heat tolerance in a population that evolved under milder conditions. However, increased heat tolerance has negative impacts for other traits: heat‐tolerant individuals carrying *ht* spend longer in exposed environments that heat‐susceptible individuals tend to avoid through their inclination to seek out shade. This increases *ht* individuals’ risk of predation (e.g., Berticat et al. 2004; Velduis et al. 2020). Increased predation risk is an indirect, negative pleiotropic effect of *ht* and offsets fitness benefits of increased heat tolerance. Thus, spread of *ht* is impeded, and so is adaptation of the population to the changing climate.

Now consider a plastic trait that mitigates the increased predation risk of *ht‐*carrying individuals: antipredator cautiousness (*pc*). Antipredator behaviors show within‐ and between‐population variation and are influenced by genes, environmental features, and their interactions (Dingemanse et al. 2019). Individuals often tune risk‐avoidance behavior to different environments, owing to differences in predator abundance (Tibblin et al. 2020). Although heightened cautiousness is beneficial in predator‐abundant habitat, it is costly in a safe environment, so environmental cue‐based reaction norms will be favored.

If *ht* is expressed in an individual that also carries an allele enhancing plastic responses to predator cues, *pc*, then its fitness benefits will outweigh negative pleiotropic effects. This single‐locus parameterization of enhanced plasticity is likely reductive, but adopted for simplicity. The pre‐existing plasticity associated with *pc* facilitates the spread of *ht*, but given both traits are genetically variable, what maintains the association between *ht* and *pc* when recombination and random assortment should break it down? One possibility is that the alleles come to be in linkage disequilibrium through greater fitness of individuals that carry both sets of traits. Alternatively, if statistical linkage is caused by physical linkage in the first place, for example proximate chromosomal locations, or co‐occurrence in a region of low recombination such as an inversion, then the phenotypic association between thermal tolerance and antipredator plasticity may interact in driving adaptation to climatic conditions. Such physical linkage may be more likely if the two traits have historically been under joint selection (Saltz et al. 2017).

To explore the importance of genetic coupling between adaptive mutations and pre‐existing plasticity to evolutionary dynamics, we simulated the above scenario in SLiM (Haller and Messer 2019). This two‐locus model is a simplification that may be expected to exaggerate linkage effects, but usefully illustrates the key importance of genetic covariance regardless of its causation. We start with two populations, each containing 500 individuals and subject to increased environmental temperature: population LP experiences low levels of predation, whereas population HP suffers greater predation. The two populations represent conditions of spatial and temporal variation in predation pressure that might historically create and maintain plasticity in antipredator cautiousness, and we assume high levels of reciprocal migration (0.5 in both directions). Thus, the parameterization of separate populations serves to enforce variable selection regimes, but the populations themselves are genetically admixed. An allele underlying the plastic trait (*pc*) is present in the metapopulation at some starting frequency and confers a fitness advantage in HP, but a weak fitness cost in LP (Table 2).

At the start of the simulation a mutation, *ht*, arises in a single diploid individual and directly benefits survival in both populations. However, *ht* indirectly increases predation risk, neutralizing net fitness benefit in HP, and reducing it in the LP population. If individuals co‐express *ht* and *pc* then they retain some net fitness benefit in the HP population, and have twice the net fitness benefit in the LP population (Table 2). Both mutations are treated as additive. Simulations were run for 1,000 generations. To evaluate the effects of linkage, both alleles were constrained to the same linkage group (i.e., chromosome) 40 kb apart, whereas to simulate independent segregation, each allele was present in a different linkage group. In both conditions *ht* initially arises in an individual carrying *pc*, with the probability of recombination between adjacent positions in linkage groups specified as 1e‐8.

**Figure 3:**
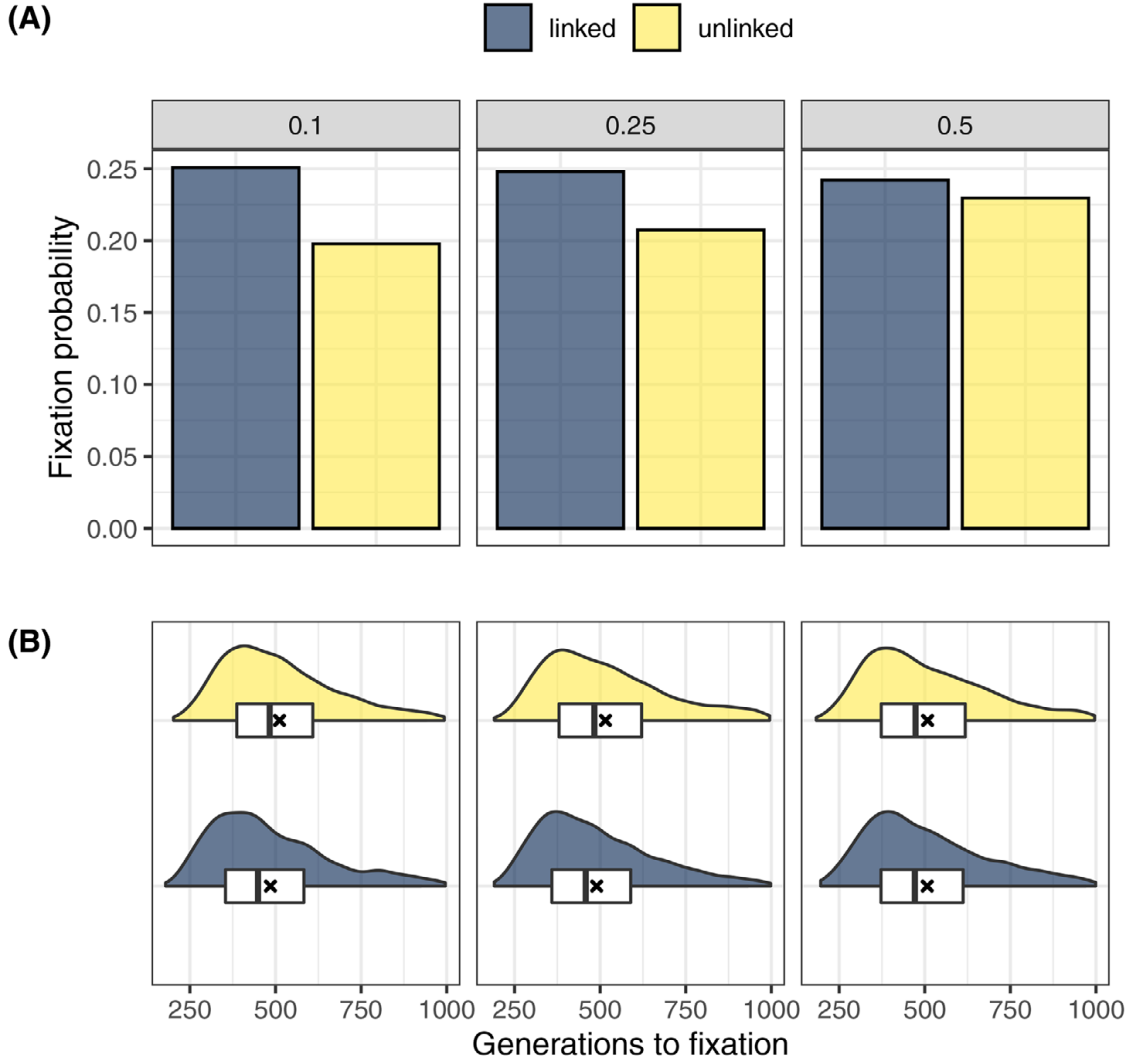
Simulation results illustrating importance of genetic covariance between an adaptive trait and plasticity that buffers its negative effects. Simulations of adaptive dynamics following emergence of an adaptive mutation with negative pleiotropic effects that can be offset by an allele that causes facilitating plasticity *pc* (itself with starting frequencies of 0.1, 0.25, and 0.5). (A) Fixation of the pleiotropic mutation is *more likely* when linked with the facilitating plasticity allele, and (B) fixation also tends to occur *more rapidly* under linkage. However, the importance of linkage to the rate of adaptation is reduced when the *pc* allele starts at a higher frequency, in which case the rate of adaptation is uniformly increased. Horizontal lines show medians, crosses means, and boxes the interquartile ranges.

Results of simulations under different *pc* starting frequencies and linkage scenarios, with 10,000 runs each, are given in Figure 3 and show that linkage between alleles had a substantial positive impact on the likelihood of fixation, and upon the number of generations until fixation, of *ht*. This was particularly the case at lower starting frequencies of *pc*. The importance of the starting frequency of the plastic allele in modulating the importance of linkage is twofold. First, if the plastic trait is already common in the population, linkage is relatively unimportant as *ht* is likely to be co‐expressed with *pc* regardless. Second, when *pc* is rare, both alleles are under strong positive selection through their combined effects on fitness. Note however that as the starting frequency of *pc* approaches zero, the probability of the adaptive mutation arising in the same genome becomes vanishingly unlikely.

**Table 2:** Fitness effects of plastic predator cautiousness (*pc*) and heat tolerance (*ht*) alleles, independently and in combination, in different predation scenarios

	*pc*	*ht* ^1^		*ht* + *pc*
		*ht* (+) direct benefits	*ht* (–) indirect costs	
LP (low predation)	–0.05	+0.2	–0.1	+0.2
HP (high predation)	+0.1	+0.2	–0.2	+0.1

^1^ *ht* fitness effects are split into direct fitness benefits (+) and indirect fitness costs (–).

## AUTHOR CONTRIBUTIONS

All authors made significant contributions to conceptual development and writing of the manuscript. JGR performed simulations. NWB led the writing.

## DATA ARCHIVING

No data are associated with this manuscript.

## CONFLICT OF INTEREST

The authors declare no conflict of interest.
